# Supplementary material for: Effects of Elevation and Distance from Highway on the Abundance and Community Structure of Bacteria in Soil along Qinghai-Tibet Highway
Source: Int J Environ Res Public Health. 2021 Dec 13;18(24):13137. doi: 10.3390/ijerph182413137 (PMC8701971; doi:10.3390/ijerph182413137)
Supplement: Supplementary file 1 [file ijerph-18-13137-s001.zip › ijerph-1473773-supplementary.pdf]

Effects of Elevation and Distance from Highway on the Abundance and Community Structure of Bacteria in Soil along Qinghai-Tibet Highway

Zhuocheng Liu [1,2], Yangang Yang [2], Shuangxuan Ji [1,2], Di Dong [1], Yinruizhi Li [1], Mengdi Wang [1], Liebao Han [1,*] and Xueping Chen [2,*]

[1] School of Grassland Science, Beijing Forestry University, Beijing, 100083, China; liuzhuocheng@bjfu.edu.cn (Z.L.); jsx11223345@163.com (S.J.); didoscori@163.com (D.D.); liyinruizhi@163.com (Y.L.); mengdi0627@163.com (M.W.);

[2] Environmental Protection and Soil and Water Conservation Research Center, China Academy of Transportation Sciences, Beijing, 100029, China; Ecologyoung@126.com (Y.Y.)

* Correspondence: hanliebao@163.com; chenxueping@vip.sina.com

Table S1 The relative abundance of the top 10 dominant bacteria in the phylum level abundance at different altitudes and distances

Sampling site	Proteobacteria	Actinobacteria	Bacteroidetes	Firmicutes	Gemmatimonadetes	Acidobacteria	Nitrospirae	Chloroflexi	Cyanobacteria	Chlorobi
L1	35.21±2.981	20.409±1.466	14.932±0.626	15.087±1.821	8.097±0.44	4.074±0.423	0.185±0.022	0.32±0.051	0.236±0.123	0.338±0.056
L2	42.021±3.514	**15.284±2.116***	**23.15±5.859***	9.724±1.671	5.625±1.429	**2.237±0.664***	0.148±0.065	0.231±0.028	0.277±0.081	0.301±0.087
L3	30.334±5.111	25.705±4.297	15.546±1.867	19.031±4.358	4.883±0.515	**2.335±0.249***	0.148±0.055	0.18±0.04	**1.084±0.535***	0.14±0.028
L4	31.883±2.654	28.769±4.468	15.866±1.88	13.57±1.837	5.616±0.467	2.513±0.204	0.172±0.023	0.279±0.038	0.223±0.046	0.29±0.053
L5	33.466±1.386	**28.876±2.005***	**13.159±0.714***	12.38±1.434	5.51±0.891	**5.057±1.414***	0.258±0.083	0.244±0.071	**0.212±0.014***	0.228±0.096
M1	33.23±1.113	31.086±1.571	14.984±0.429	11.232±1.402	5.763±0.788	2.212±0.062	0.36±0.007	**0.322±0.042***	0.212±0.023	0.18±0.029
M2	31.536±2.033	24.905±2.06	20.382±1.251	14.764±1.714	5.154±0.968	1.868±0.163	0.236±0.087	0.185±0.055	0.177±0.037	0.266±0.067
M3	**28.025±5.99***	28.665±3.141	19.014±3.717	16.48±5.286	5.037±0.987	1.388±0.262	0.314±0.139	0.247±0.044	0.169±0.046	0.236±0.075
M4	32.279±1.643	28.022±2.218	16.86±0.749	12.652±0.629	5.9±0.452	2.633±0.239	0.255±0.011	0.282±0.01	0.148±0.025	0.242±0.007
M5	**40.416±1.167***	21.315±1.206	15.84±0.918	15.032±2.813	3.842±0.686	2.279±0.207	0.244±0.03	**0.129±0.013***	0.25±0.035	0.164±0.071
H1	28.172±1.4	**29.191±2.925***	**19.082±0.179***	**10.61±1.476***	7.698±0.394	**2.611±0.35***	**1.131±0.389***	**0.363±0.067***	0.185±0.027	**0.47±0.049***
H2	23.463±1.324	**30.857±3.883***	22.241±2.703	**15.132±2.567***	4.34±0.405	1.946±0.228	0.837±0.06	0.306±0.03	0.161±0.034	0.346±0.044
H3	20.827±5.252	15.411±4.685	30.366±4.974	27.989±6.144	2.768±0.733	1.522±0.319	0.303±0.086	0.15±0.055	0.164±0.031	0.134±0.05
H4	23.612±1.236	25.079±0.976	22.7±1.323	17.975±1.923	4.432±0.569	**3.306±0.562***	0.908±0.107	**0.865±0.137***	0.148±0.019	0.309±0.034
H5	24.426±1.779	**15.551±1.501***	**29.278±2.596***	**26.195±0.822***	1.613±0.146	**1.388±0.257***	**0.494±0.077***	**0.148±0.043***	0.191±0.017	**0.153±0.025***

* indicate significant difference between treatments and control(400m) at $p < 0.05$.

Table S2 Adonis analysis in 5m,20m,50m,100m and 400m from the highway for plant and soil bacterial respectively in 3sites and adonis analysis in altitudes of 4000m,4600m and 5200m for plant and soil bacterial

Community	R^2	F	p
Plant community in 4000m	0.71	9.26	0.001
Plant community in 4600m	0.70	8.68	0.001
Plant community in 5200m	0.49	3.58	0.001
Bacterial community in 4000m	0.36	2.19	0.001
Bacterial community in 4600m	0.43	2.89	0.001
Bacterial community in 5200m	0.52	4.05	0.001
Bacterial community in 3 sites	0.31	12.66	0.001
Plant community in 3 sites	0.28	11.03	0.001
